# Supplementary material for: One-hour extraction-free loop-mediated isothermal amplification HPV DNA assay for point-of-care testing in Maputo, Mozambique
Source: Nat Commun. 2025 Aug 7;16:7295. doi: 10.1038/s41467-025-62454-x (PMC12331945; doi:10.1038/s41467-025-62454-x)
Supplement: Supplementary file 1 — Supplementary Information [file 41467_2025_62454_MOESM1_ESM.pdf]

Supplementary Figures

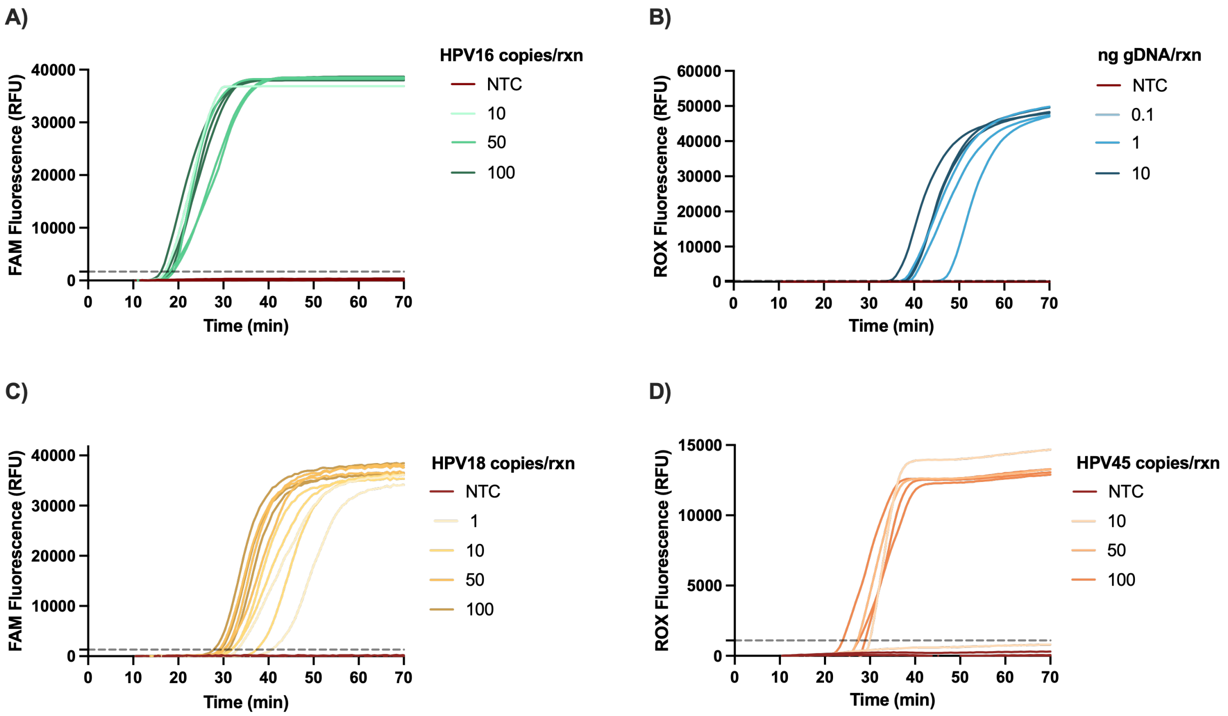

Supplementary Figure 1. Amplification curves for the singleplex DARQ LAMP reactions performed on the Bio-Rad thermocycler with a range of input copies of target. These results are for the **A)** HPV16, **B)** gDNA, **C)** HPV18, and **D)** HPV45 reactions. n=3 replicates. Source data are provided as a Source Data file.

Optimization of the multiplexed HPV16/gDNA reaction

In order to develop the final multiplexed reactions, concentrations of individual reagents were first optimized. Raw data is shown in all HPV16/gDNA optimization experiments in order to portray the differences caused by different concentrations of reagents. It was previously demonstrated that higher proportions of labeled FIP to unlabeled FIP lead to higher fluorescence outputs but could also increase time to detection[1]. Therefore, we began by evaluating the effect of different ratios of labeled to unlabeled FIP in the gDNA and HPV16 reactions performed on the Bio-Rad. The time to detection increased with higher amounts of labeled FIP in the gDNA reaction (Supplementary Figure 2A); however, the differences were notably smaller for the HPV16 reaction (Supplementary Figure 2B).

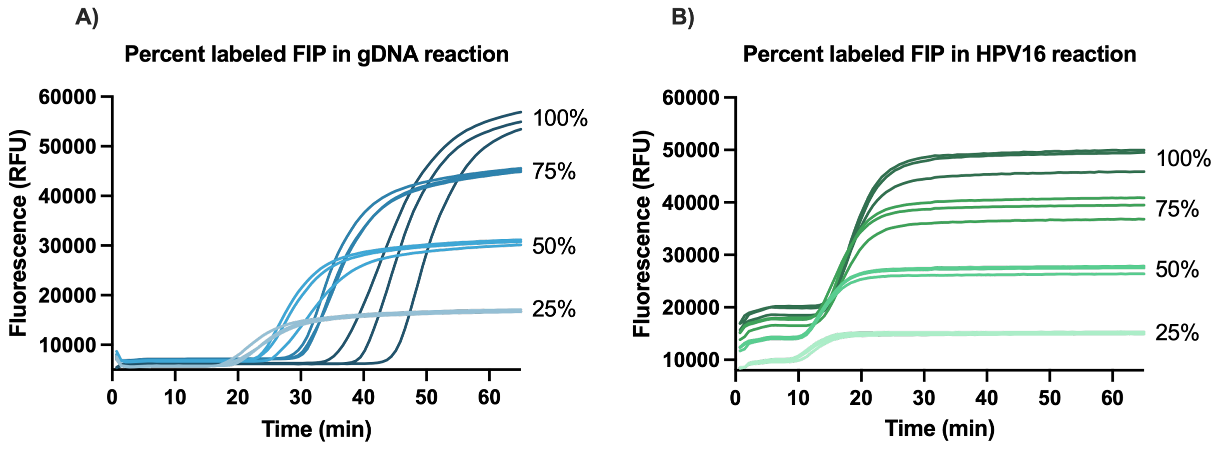

Supplementary Figure 2. Effect of different percentages of labeled FIP on time to detection and total fluorescence on the Bio-Rad thermocycler. A) gDNA singleplex reaction read on ROX channel with 10 ng gDNA/reaction. **B)** HPV16 singleplex reaction read on FAM channel with 100 copies HPV16 DNA/reaction. Raw data shown. n=3 replicates. Source data are provided as a Source Data file.

We then tested the unoptimized HPV16/gDNA multiplexed assay on our target device, the Axxin T8-ISO. In the multiplexed reaction, we labeled 5%, 7% or 10% of the HPV16 FIP and 25% of the gDNA FIP (Supplementary Figure 3). With 5% labeled FIP very low fluorescence signal was observed (Supplementary Figure 3A), whereas with 10%, the FAM (HPV16) channel immediately saturated (Supplementary Figure S3C). With 7% labeled HPV16 FIP, clear amplification was observed (Supplementary Figure 3B), so future experiments were conducted with this concentration. For the gDNA reaction, a rapid, clear increase in fluorescence signal was produced with 25% labeled gDNA FIP (Supplementary Figure 2D), so this percentage was selected for gDNA for subsequent optimization experiments.

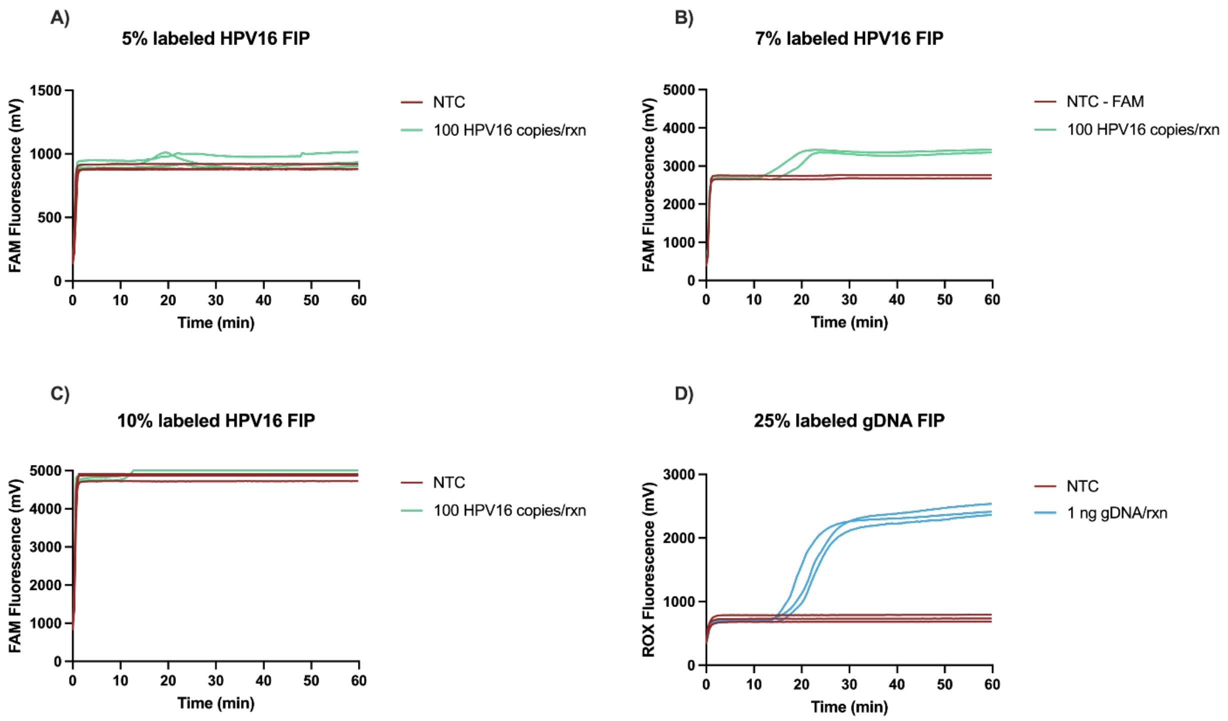

Supplementary Figure 3. Effect of different percentages of labeled FIP in HPV16/gDNA multiplexed reaction on assay readout on Axxin T8-ISO. A) 5% labeled HPV16 FIP read on FAM channel (n=3 replicates) **B)** 7% labeled HPV16 FIP read on FAM channel (n=2 replicates). This was the chosen percentage of labeled HPV16 FIP for future experiments. **C)** 10% labeled HPV16 FIP read on FAM channel (n=3 replicates) **D)** 25% labeled gDNA FIP read on ROX channel (n=3). Raw data shown. *NTC = no target control.* Source data are provided as a Source Data file.

Next, concentrations of magnesium sulfate ($MgSO_4$, Supplementary Figure 4) and betaine (Supplementary Figure 5) were optimized in the multiplexed reaction on the Bio-Rad. $MgSO_4$ concentration was optimized first, and the selected concentration of 8 mM was used in the betaine optimization experiment. The optimal concentrations selected for the shortest time to detection and consistent amplification were 8 mM $MgSO_4$ and 0.4 M betaine.

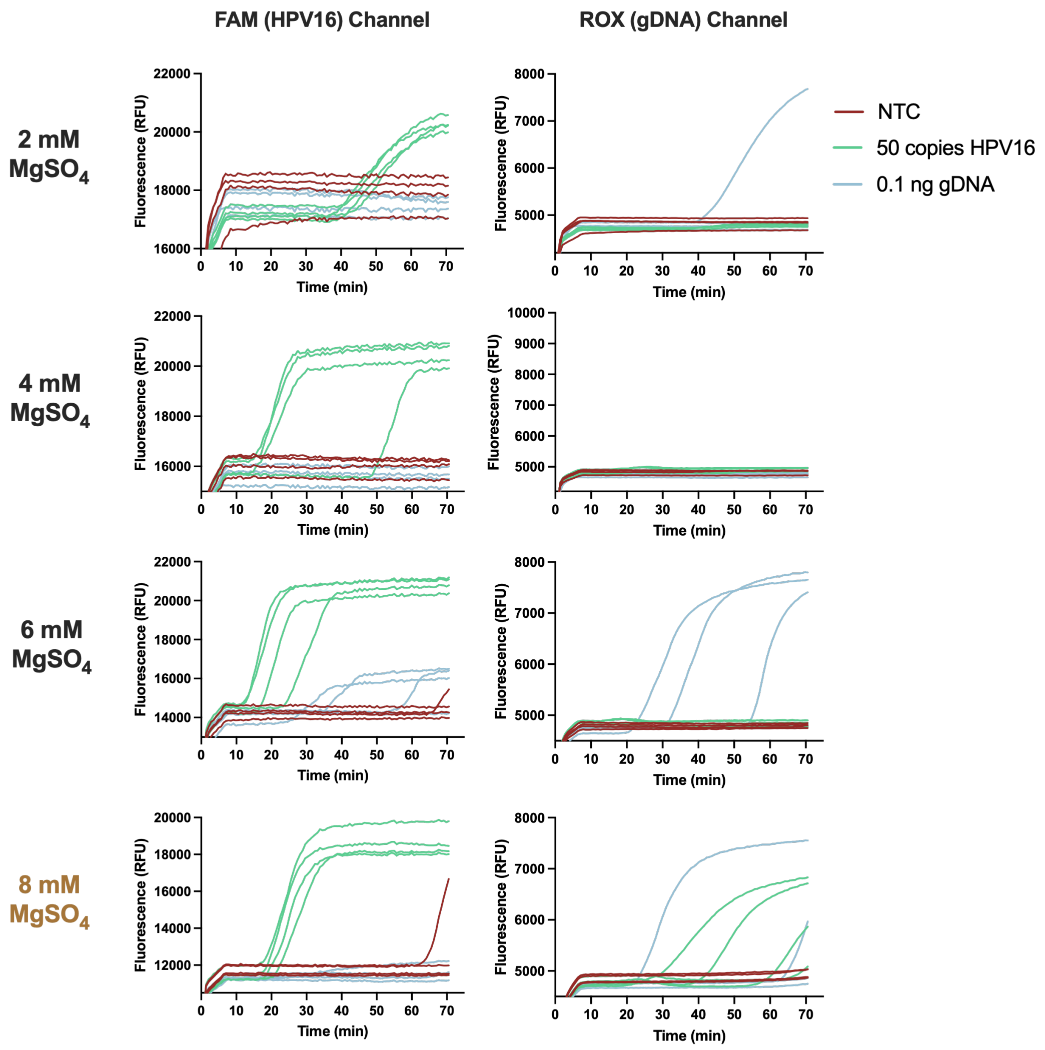

Supplementary Figure 4. Optimization of the concentration of $MgSO_4$ in the multiplexed HPV16/gDNA reaction on the Bio-Rad thermocycler. gDNA and SiHa DNA were used as targets in each condition in concentrations equivalent to 0.1 ng/reaction of gDNA and 50 copies/reaction of HPV16, respectively. Because DNA extracted from SiHa cells includes human genomic and HPV16 DNA, SiHa amplification is expected in both the FAM and ROX channels, but gDNA amplification is only expected in the ROX channel. With increasing concentrations of $MgSO_4$, noise is reduced and more consistent amplification is observed. With 6 mM $MgSO_4$, unexpected fluorescence in the FAM channel was produced with gDNA as target. However, this was not present at 8 mM $MgSO_4$, the selected concentration for the multiplexed reaction. Raw data shown. *NTC = no target control.* n=4 replicates. Source data are provided as a Source Data file.

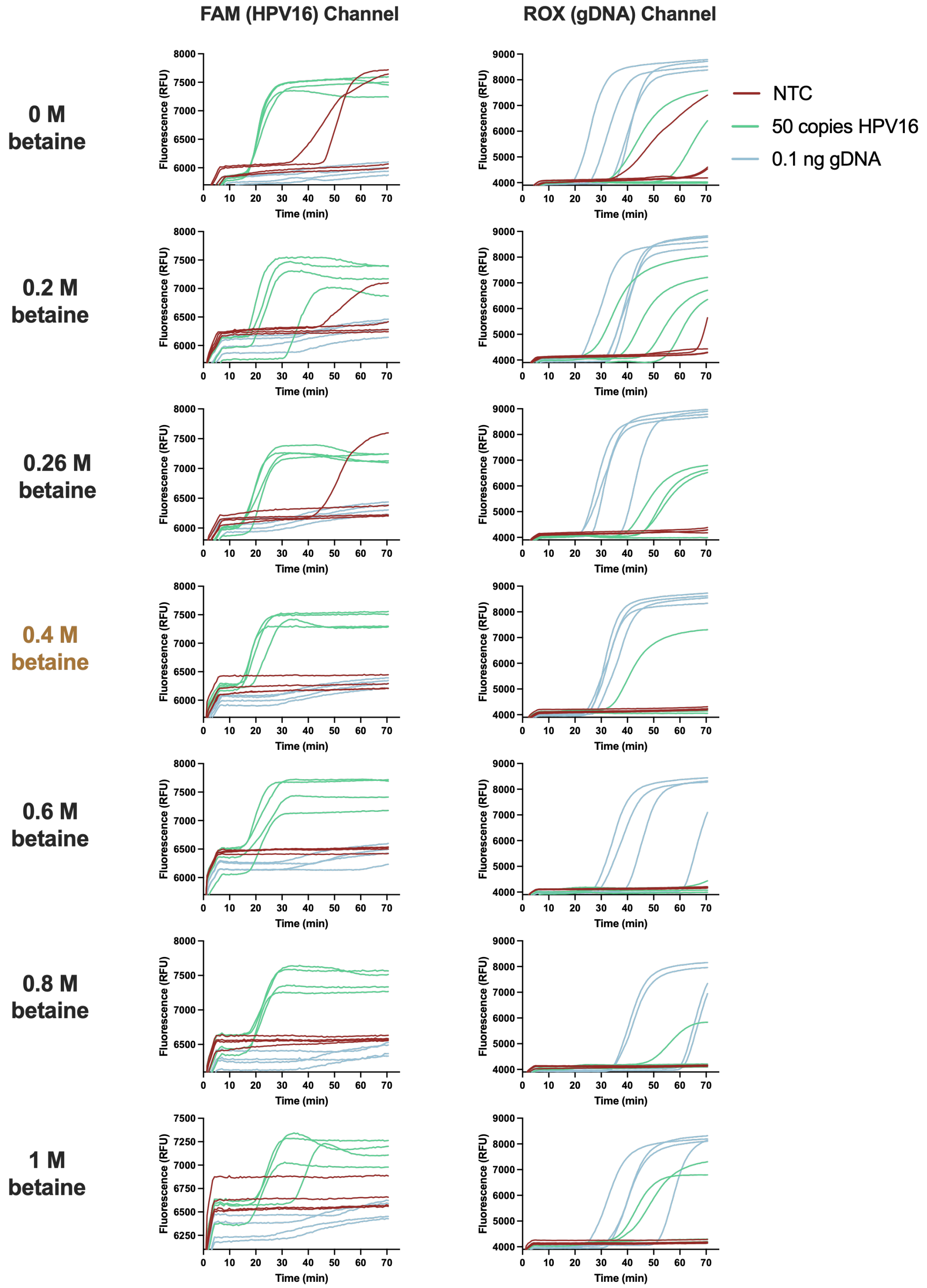

Supplementary Figure 5. Optimization of the concentration of betaine in the multiplexed HPV16/gDNA reaction on the Bio-Rad thermocycler. gDNA and DNA extracted from SiHa cells were used as the targets in each condition in concentrations equivalent to 0.1 ng/reaction of gDNA and 50 copies/reaction of HPV16, respectively. Because DNA extracted from SiHa cells includes human genomic and HPV16 DNA, SiHa amplification is expected in both the FAM and ROX channels, but gDNA amplification is only expected in the ROX channel. A few no target control (NTC) replicates amplified with betaine concentrations below 0.4 M, but none did at higher concentrations. 0.4 M produced the fastest and most consistent amplification and was chosen for the optimized reaction. Raw data shown. *NTC = no target control*. n=4 replicates. Source data are provided as a Source Data file.

Lastly, the optimized reaction was re-tested on the T8-ISO with either 7% or 25% labeled gDNA FIP (Supplementary Figure 6). As expected, with 25% of FIP labeled, more fluorescence was produced, but slightly faster amplification was produced with 7% of FIP labeled. We thus proceeded with 7% labeled FIP for both HPV16 and gDNA in the optimized multiplexed reaction.

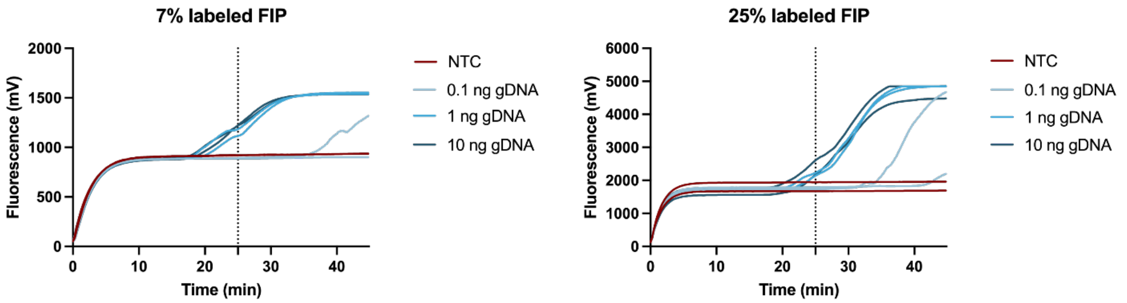

Supplementary Figure 6. Comparison of 7% and 25% labeled gDNA FIP in the multiplexed HPV16/gDNA reaction on the Axxin T8-ISO. At 25 minutes, amplification was more evident with 7% labeled FIP, which was chosen for the final optimized reaction. Raw data shown. *NTC = no target control*. n=2 replicates. Source data are provided as a Source Data file.

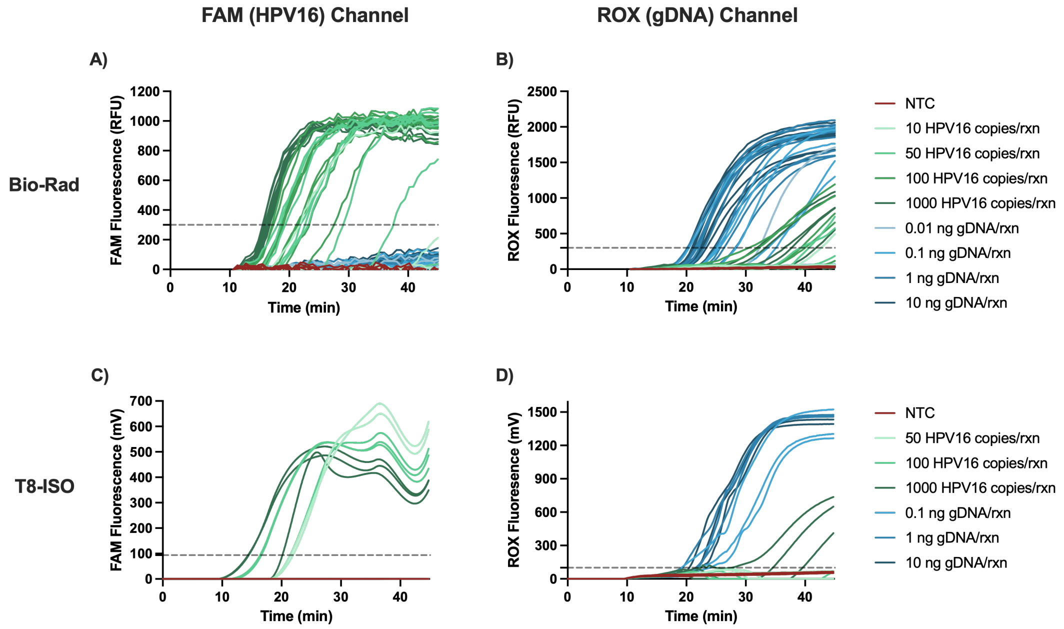

Supplementary Figure 7. Amplification curves for the multiplexed HPV16/gDNA DARQ LAMP reaction. The reaction was performed on the Bio-Rad **(A&B)** and read on the **A)** FAM channel for HPV16 detection (n=9 replicates) and **B)** ROX channel for gDNA detection (n=9 replicates). The reaction was also performed on the T8-ISO **(C&D)** and read on the **C)** FAM channel (n=3 replicates) and **B)** ROX channel (n=3 replicates). As expected, on both devices some replicates with DNA extracted from SiHa cells amplified on the ROX (gDNA) channel because SiHa cells contain both human genomic DNA and HPV16 DNA. The reactions with SiHa DNA consistently produced signal in the HPV16 (FAM) channel but not always in the gDNA (ROX) channel, indicating that the HPV16 primers dominate in the multiplexed reaction when SiHa DNA is used as the sample. Source data are provided as a Source Data file.

Optimization of the multiplexed HPV18/45 reaction

For the multiplexed HPV18/45 reaction with DNA-intercalating fluorescent dye, concentrations of $MgSO_4$, betaine, and polymerase were optimized sequentially on the Bio-Rad (Supplementary Figure 8). The optimal concentration of $MgSO_4$ was 6 mM, which provided the most consistent and fastest amplification (Supplementary Figure 8A), and was used for subsequent experiments. Interestingly, betaine negatively impacted this reaction in distinct ways for each target (Supplementary Figure 8B). For HPV18, time to detection consistently increased as betaine concentration increased. For HPV45, the variability in time to detection between replicates increased as betaine concentration increased. Therefore, betaine was henceforth omitted from this reaction. Lastly, the concentration of polymerase was optimized. While little difference was observed in the amplification of HPV18 DNA, doubling the concentration of polymerase allowed for faster and more consistent amplification of HPV45 DNA (Supplementary Figure 8C).

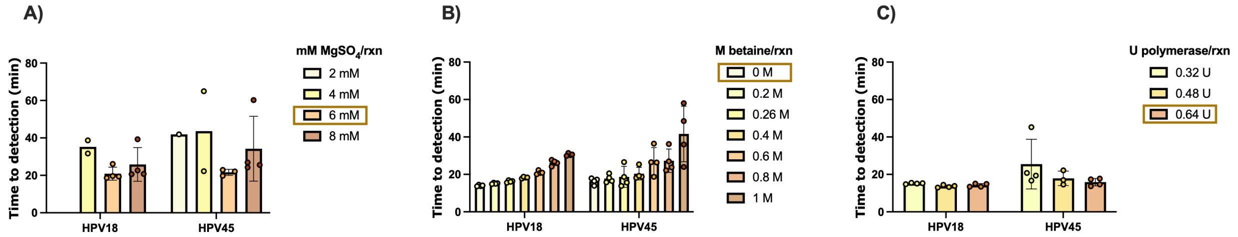

Supplementary Figure 8. Optimization of reagents in the multiplexed HPV18/45 reaction on the Bio-Rad thermocycler. HeLa and MS751 DNA were used as the targets in each condition in concentrations equivalent to 50 copies/reaction of HPV18 and HPV45, respectively. Concentrations of **A)** magnesium sulfate ($MgSO_4$), **B)** betaine, and **C)** DNA polymerase were optimized. The selected concentrations are boxed in the figure legends. The DNA-intercalating dye produces fluorescence in the FAM channel. Data were baseline subtracted and corrected for fluorescence drift, and a single positivity threshold was set automatically by the instrument software. Time to detection was determined from the time at which fluorescence meets the positivity threshold. Bar graphs represent mean time to detection and error bars represent standard deviation. n=4 replicates. Source data are provided as a Source Data file.

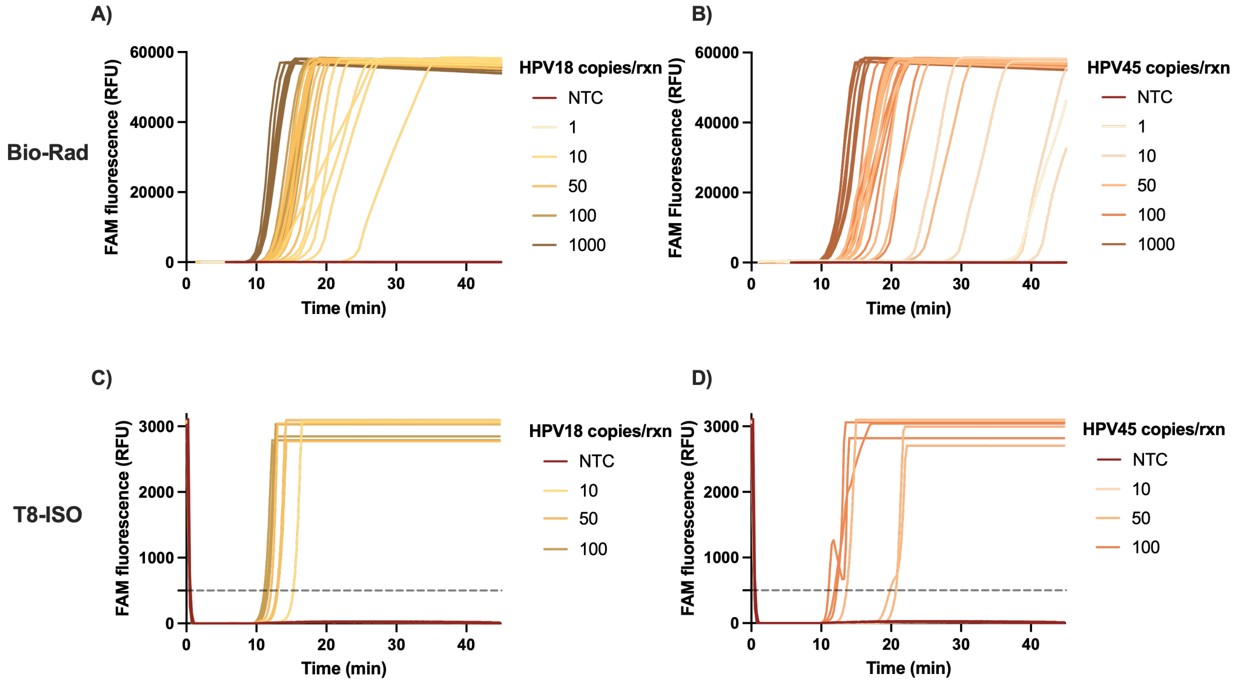

Supplementary Figure 9. Amplification curves for the multiplexed HPV18/45 reaction. The reaction was performed on the Bio-Rad **(A&B)** and read on the FAM channel with **A)** HPV18 target (n=9 replicates) and **B)** HPV45 target (n=9 replicates). The reaction was also performed on the T8-ISO **(C&D)** and read on the FAM channel with **C)** HPV18 target (n=3 replicates) and **D)** HPV45 target (n=3 replicates). Source data are provided as a Source Data file.

Specificity

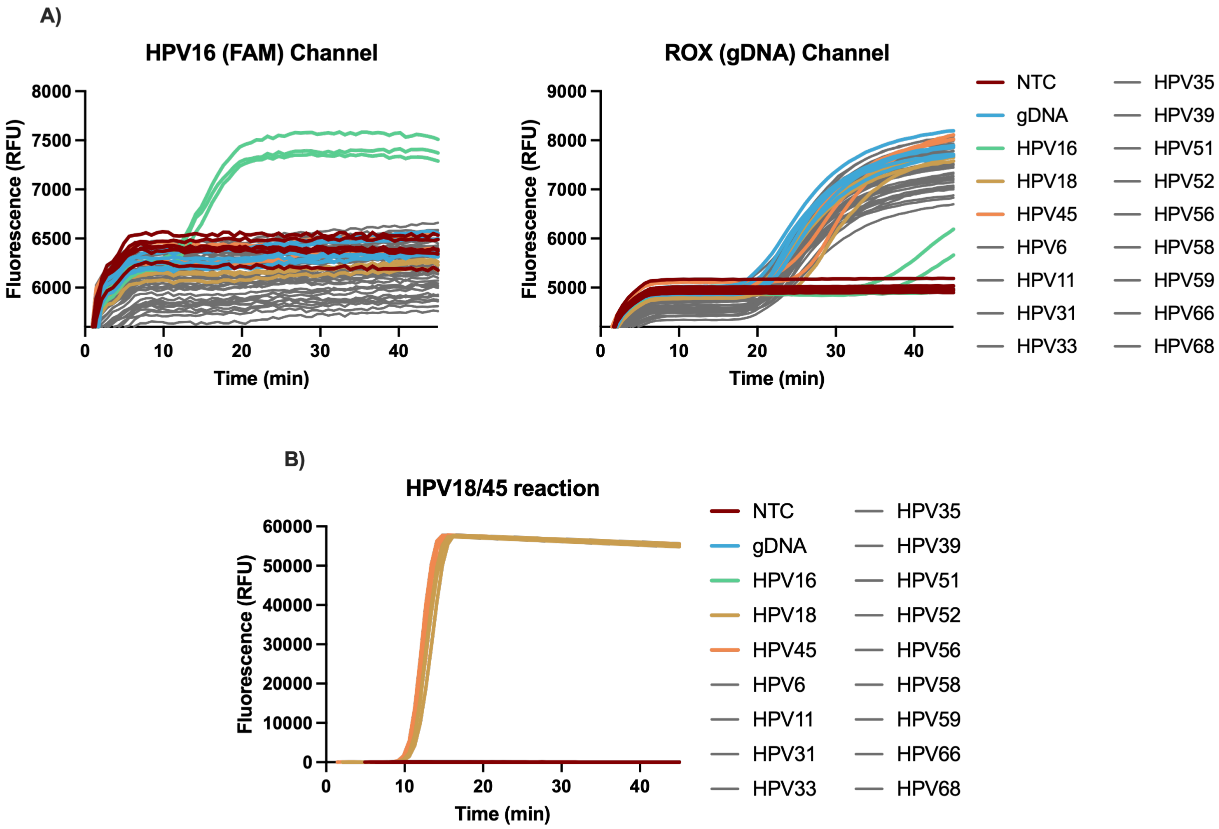

Supplementary Figure 10. Specificity of the multiplexed assays on the Bio-Rad thermocycler. For each HPV type, 10^6 copies/reaction in a background of 10 ng gDNA/reaction were used. **A)** HPV16/gDNA reaction with n=3 replicates for each HPV type, n=6 replicates for gDNA, and n=6 replicates for NTCs. Only the HPV16 sample amplified in the FAM channel, whereas all samples amplified in the ROX channel due to the background of gDNA. Raw data shown. **B)** HPV18/45 reaction with n=3 replicates for each HPV type, n=3 replicates for gDNA, and n=6 replicates for NTCs. Data were baseline subtracted and corrected for fluorescence drift by the Bio-Rad software. Source data are provided as a Source Data file.

Sample-to-answer workflow

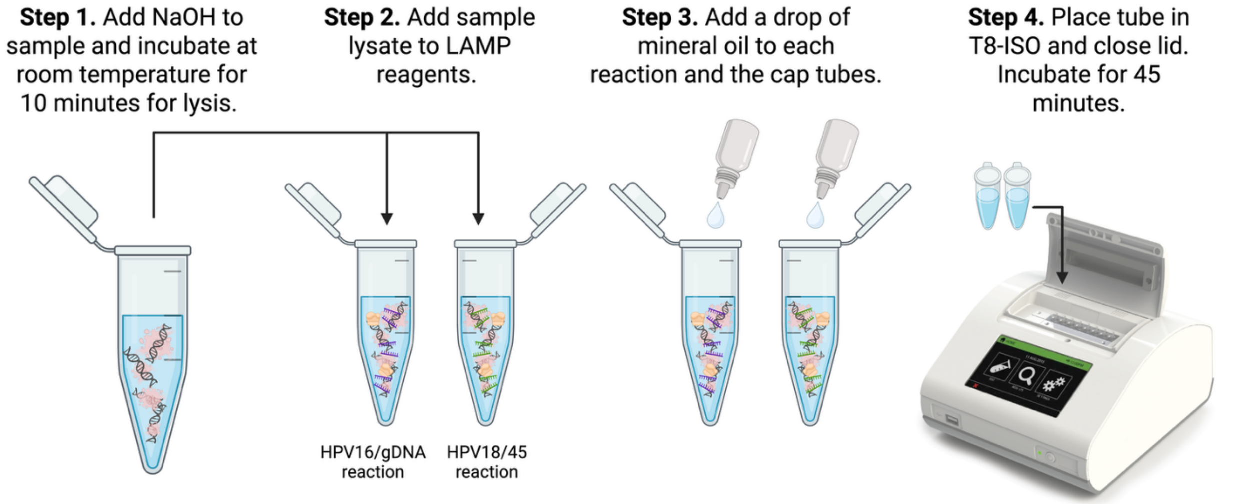

Supplementary Figure 11. Representation of the extraction-free workflow used for validating the HPV LAMP assay with cultured cells and clinical samples. Created in BioRender. Barra, M. (2025) https://BioRender.com/42rvoie.

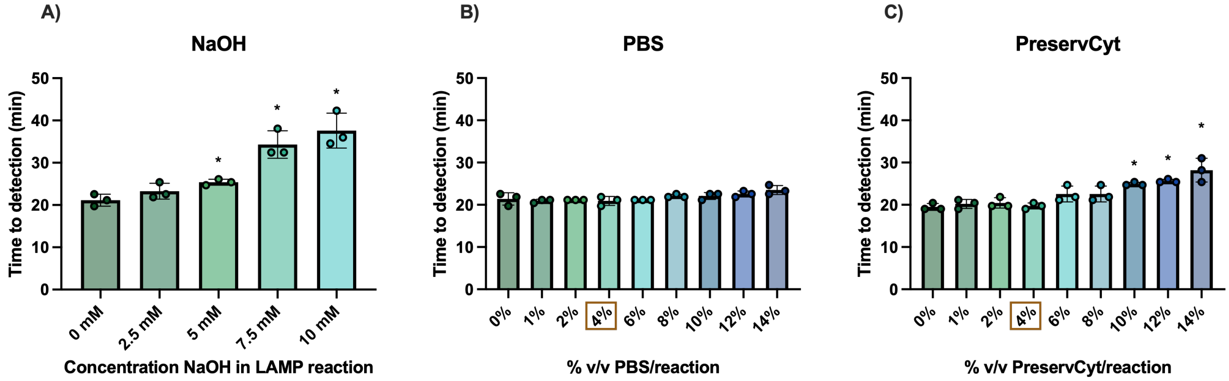

Supplementary Figure 12. Tolerance of HPV16/gDNA reaction for different additives on the Bio-Rad thermocycler. 1 ng gDNA/reaction was used as the target. Changes in time to detection were evaluated under different concentrations of additives. Statistically significant difference in mean time to detection between the base condition (no additive) and other conditions was analyzed with an unpaired two-sided Student's t-test assuming Gaussian distribution and a significance level of <0.05 (*, $p<0.05$). **A)** NaOH: For cell lysis 1 M NaOH was used which corresponds to 4 mM NaOH in the LAMP reaction. p=0.0097, 0.0030, and 0.0028 for 5 mM, 7.5 mM, and 10 mM, respectively. **B)** Phosphate buffered saline (PBS): After lysing clinical samples collected in Houston, an equivalent of 4% v/v PBS/reaction is added to the LAMP reaction. **C)** PreservCyt: After lysing samples collected in Mozambique, an equivalent of 4% v/v PreservCyt/reaction is added to the LAMP reaction. p=0.0005, 0.0003, and 0.0068 for 10%, 12%, and 14%, respectively. Bar graphs represent mean time to detection and error bars represent standard deviation. n=3 replicates. Source data are provided as a Source Data file.

Clinical sample testing

To clinically validate a novel HPV DNA test for cervical cancer screening, comparison of HPV results with histological analysis is required. While HPV DNA testing is a sensitive biomarker for cervical cancer screening, it is not considered a specific biomarker, as HPV infections can be cleared naturally before becoming cancerous[2,3]. Cervical intraepithelial neoplasia (CIN) grades 2 and 3 are precancers that can turn into invasive cervical cancer if not treated[4]. Histological analysis was performed on 19/38 of the Houston samples. While this dataset is insufficient for formal clinical validation, it is notable that four out of five samples positive for HPV16 or HPV18/45 were associated with histologically confirmed CIN2+ lesions and none of the 14 HPV 16/18/45 negative samples were associated with CIN2+ lesions.

Supplementary Table 1. Comparison of GeneXpert, LAMP and Histology Results for Samples Collected in Houston with Histology Results Available

Sample number	GeneXpert	LAMP	Histology	Provider/ self collected	Collection buffer
1	neg	neg	Normal	Provider	PBS
2	neg	neg	Normal	Provider	PBS
3	neg	neg	Normal	Provider	PBS
4	neg	neg	Normal	Self	PBS
5	neg	neg	Normal	Provider	PBS
6	neg	neg	Normal	Provider	PBS
7	neg	neg	CIN1	Provider	PBS
8	neg	neg	CIN1	Provider	PBS
9	neg	neg	CIN1	Self	PBS
10	neg	neg	CIN1	Provider	PBS
11	neg	neg	CIN1	Self	Water
12	P3	neg	Normal	Provider	PBS
13	P5	neg	Normal	Provider	PBS
14	P4	neg	CIN1	Provider	PBS
15	HPV16	HPV16	**VAIN2/3***	Provider	PBS
16	HPV16	HPV16	**CIN2**	Provider	PBS
17	HPV16	HPV16	**CIN2/3**	Self	PBS
18	HPV18/45, P3	HPV18/45	CIN1	Self	Water
19	HPV18/45	HPV18/45	**CIN3**	Self	Water

CIN: cervical intraepithelial neoplasia; *VAIN*: vaginal intraepithelial neoplasia
PBS: phosphate buffered saline
P3: HPV31/33/35/52/58 positive on GeneXpert
P4: HPV51/69 positive on GeneXpert
P5: HPV39/56/66/68 positive on GeneXpert
*Clinical note: "Fragments of endocervical and squamous epithelium with metaplastic changes"

Supplementary Table 2. Comparison of GeneXpert, LAMP, and Histology Results for Samples Collected in Houston at Visits Where Histology Was Not Performed

Sample number	GeneXpert	LAMP	Histology	Provider/ self collected	Collection buffer
20	P4/P5	neg	N/A	Provider	PBS
21	P3	neg	N/A	Provider	PBS
22	neg	neg	N/A	Provider	PBS
23	neg	neg	N/A	Provider	PBS
24	neg	neg	N/A	Provider	PBS
25	neg	neg	N/A	Provider	PBS
26	neg	neg	N/A	Provider	PBS
27	neg	neg	N/A	Provider	PBS
28	neg	neg	N/A	Provider	PBS
29	neg	neg	N/A	Provider	PBS
30	neg	neg	N/A	Provider	PBS
31	neg	neg	N/A	Provider	PBS
32	neg	neg	N/A	Provider	PBS
33	neg	neg	N/A	Provider	PBS
34	neg	neg	N/A	Provider	PBS
35	HPV16	HPV16	N/A	Provider	PBS
36	HPV16	HPV16	N/A	Provider	PBS
37	HPV18/45	HPV18/45	N/A	Provider	PBS
38	HPV18/45	HPV18/45	N/A	Provider	PBS

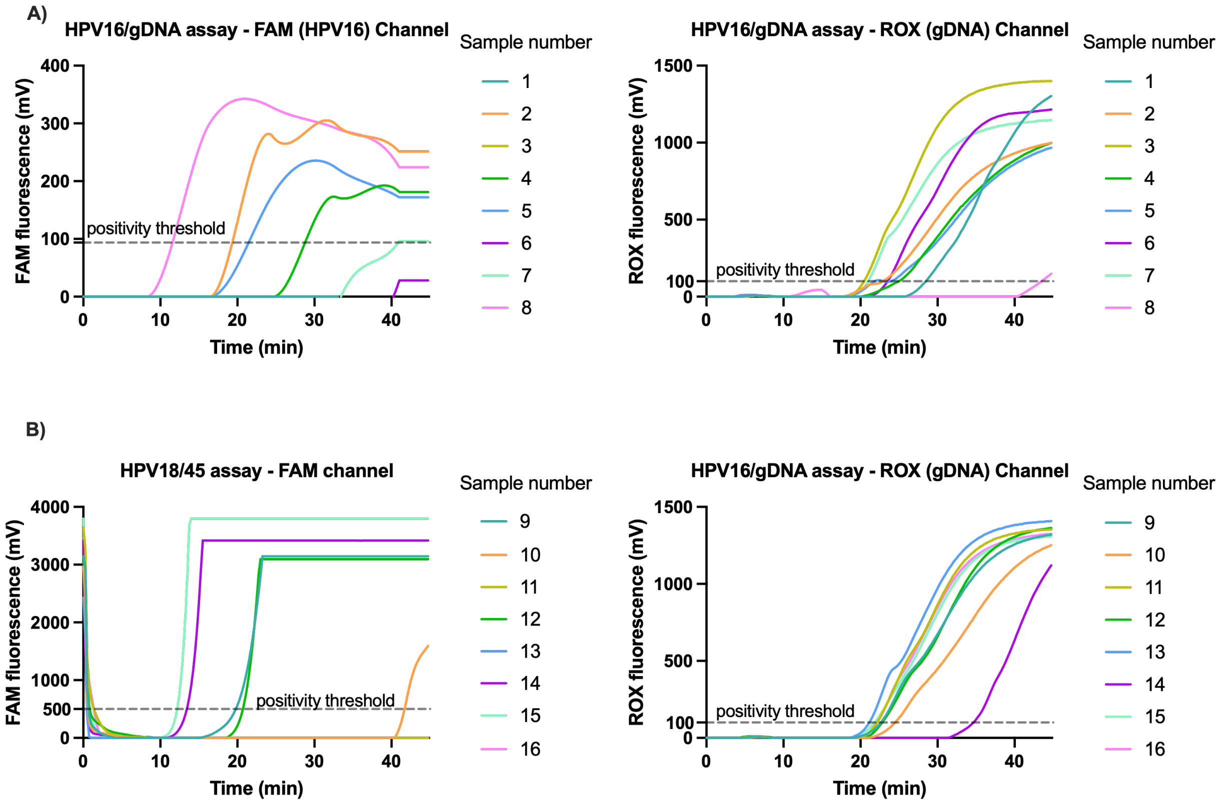

Supplementary Figure 13. Example of amplification curves for clinical samples on the Axxin T8-ISO. These are the amplification curves for 16 of the 191 samples tested in Mozambique. **A)** Amplification curves from the HPV16/gDNA reaction on the FAM (HPV16) channel (left) and ROX (gDNA) channel (right). **B)** Amplification curves from the HPV18/45 reaction on the FAM channel (left) and from the HPV16/gDNA reaction for the same samples on the ROX (gDNA) channel (right). Source data are provided as a Source Data file.

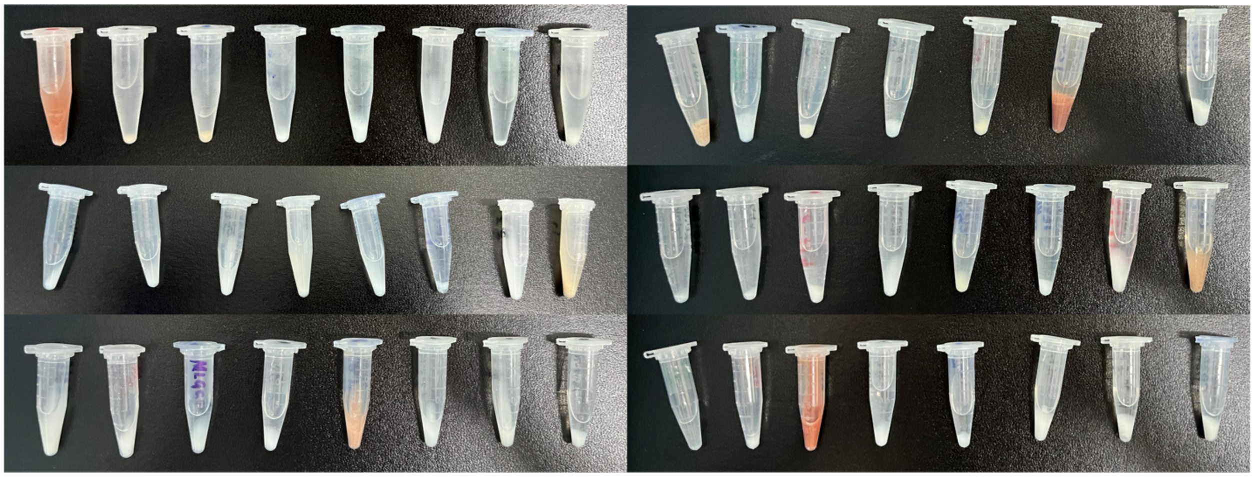

Supplementary Figure 14. Photos of a subset of the clinical samples tested in Mozambique. Variations in blood content, turbidity, and color can be observed.

Methods

Supplementary Table 3. Cost breakdown of LAMP test

**Materials**	**USD**
Puritan Flocked Swab	$0.36
Sample collection tube	$0.36
Sample collection buffer	$0.01
Reaction tubes	$0.32
Reagents	$4.06
**Total**	**$5.11**

These numbers are based on the authors' current costs and could be reduced if HPV LAMP assay kits are produced at scale.

Supplementary Table 4. Sequences for primers used in this work.

Primer	Sequence (5'→3')	Reference	Amplification method
**HPV16 fwd**	TAACCTTTTGTTGCAAGTGTGA	5	qPCR
**HPV16 rev**	TTTGTACGCACAACCGAAGC		
**HPV18 fwd**	CAACGTCACACAATGTTGTGTA		
**HPV18 rev**	TCAATTCTGGCTTCACACTTAC		
**HPV45 fwd**	GGAACCTCAGAATGAATTAGATCC		
**HPV45 rev**	TGCTCGTAACACAACAGGTCA		
**HPV16 F3**	ATGCACCAAAAGAGAACTGC	6	LAMP
**HPV16 B3**	ACAGCATATGGATTCCCATCTC		
**HPV16 FIP**	TGTTTGCAGCTCTGTGCATAATTTTGTTTCAGGACC CACAGGA		
**HPV16 BIP**	AGAATGTGTGTACTGCAAGCAATTTTATCCCGAAA AGCAAAGTCAT		
**HPV16 LF**	GTGGTAACTTTCTGGGTCGC	7	
**HPV16 LB**	AGTTACTGCGACGTGAGGT		
**HPV18 F3**	CGCGTCCTTTATCACAGG	8	
**HPV18 B3**	TGGAATCCCCATAAGGATC		
**HPV18 FIP**	GGCACCATATCCAGTATCTACCATAATTGCCCCCC TTTAGAACT		
**HPV18 BIP**	TGCAAGATACTAAATGTGAGGTACCGCAGACATTT GTAAATAATCAGGAT		
**HPV18 LF**	TCACCATCTTCCAAAACTG		
**HPV18 LB**	ATTGGATATTTGTCAGTCT		
**HPV45 F3**	CCTGTTGACCTGTTGTGTTA	9	
**HPV45 B3**	TCCGCCATTGTAGATTATTGG		
**HPV45 FIP**	GGCTGGTAGTTGTGCATGACTTTT TAGCAATTAAGCGAGTCAGAG		
**HPV45 BIP**	TGTTGTAAGTGTGACGGCAGAATTTTCTGCTGTAG TGTTCTAAGGTC		
**HPV45 LF**	AACTCCATCTGCTTCATCGTT		
**HPV45 LB**	AGTAGAGAGCTCGGCAGA		
**gDNA F3**	AGTACCCCATCGAGCACG	10	
**gDNA B3**	AGCCTGGATAGCAACGTACA		
**gDNA FIP**	GAGCCACACGCAGCTCATTGTATCACCAACTGGGA CGACA		
**gDNA BIP**	CTGAACCCCAAGGCCAACCGGCTGGGGTGTTGAA GGTC		
**gDNA LF**	TGTGGTGCCAGATTTTCTCCA		
**gDNA LB**	CGAGAAGATGACCCAGATCATGT		
**HPV16_QFIP**	/5IABkFQ/TGTTTGCAGCTCTGTGCATAATTTTGTTT CAGGACCCACAGGA	N/A	DARQ LAMP
**HPV16_Fd_FAM**	TTATGCACAGAGCTGCAAACA/36-FAM/		
**gDNA_QFIP**	/5IAbRQ/GAGCCACACGCAGCTCATTGTATCACCAA CTGGGACGACA		
**gDNA_Fd_ROX**	TACAATGAGCTGCGTGTGGCTC/3Rox_N/		
**HPV18_QFIP**	/5IABkFQ/GGCACCATATCCAGTATCTACCATAATT GCCCCCCTTTAGAACT		
**HPV18_Fd_FAM**	TATGGTAGATACTGGATATGGTGCC/36-FAM/		
**HPV45_QLF**	/5IAbRQ/AACTCCATCTGCTTCATCGTT		
**HPV45_Fd_ROX**	GAAGCAGATGGAGTT/3Rox_N/		

Integrated DNA Technologies (IDT) nomenclature is used. For DARQ LAMP, the sequences to which the probes bind are shown in blue.

Supplementary Table 5. Primer concentrations in the LAMP reactions. All concentrations are in µM.

Primer	HPV16	gDNA	HPV18	HPV45
F3	0.2	0.2	0.2	0.2
B3	0.2	0.2	0.2	0.2
FIP	1.6	1.6	1.6	1.6
BIP	1.6	1.6	1.6	1.6
LF	0.4	0.4	0.8	0.4
LB	0.4	0.4	0.8	0.4

All reactions had a total of 1.6 µM FIP. For reactions with 7% labeled FIP (QFIP:Fd), the concentration per reaction of QFIP:Fd was 0.112 µM and of FIP alone was 1.488 µM.

Supplementary Table 6. Composition of singleplex reactions.

Reagent	Volume/reaction (µL)
Nuclease-free water	10.7
Betaine (5M)	1.3
$MgSO_4$ (100 mM)	1.5
Isothermal amplification buffer (10X)	2.5
dNTP mix (10 mM)	3.5
Primer mix (10 X)	2.5
Bst 2.0 WarmStart DNA Polymerase (8000 U/ml)	2.0
Target DNA	1.0
**Total**	25

Supplementary Table 7. Composition of the optimized multiplexed reactions.

	HPV16/gDNA reaction	HPV18/45 reaction
**Reagent**	**Volume/reaction (µL)**	
Nuclease-free water	7.0	9.25
Betaine (5 M)	2.0	-
$MgSO_4$ (100 mM)	2.0	1.5
Isothermal amplification buffer (10X)	2.5	2.5
Fluorescent dye (50X)	-	0.25*
dNTP mix (10 mM)	3.5	3.5
Primer mix #1 (10X)	2.5	2.5
Primer mix #2 (10X)	2.5	2.5
Bst 2.0 WarmStart DNA Polymerase (8000 U/mL)	2.0	2.0
Target DNA	1.0	1.0
**Total**	25	25

*0.25 µL of fluorescent dye were used in all reactions run on the T8-ISO and 0.5 µL were used on reactions run on the Bio-Rad.

Additionally, one drop of mineral oil was added to the top of all reactions run on the T8-ISO. Otherwise, the reaction composition was the same when run on either instrument.

References

1. Tanner, N. A., Zhang, Y. & Evans, T. C. Simultaneous multiple target detection in real-time loop-mediated isothermal amplification. *BioTechniques* **53**, 81–89 (2012).
2. Benevolo, M. *et al.* Sensitivity, Specificity, and Clinical Value of Human Papillomavirus (HPV) E6/E7 mRNA Assay as a Triage Test for Cervical Cytology and HPV DNA Test ▿. *J. Clin. Microbiol.* **49**, 2643–2650 (2011).
3. Ramírez, A. T. *et al.* Performance of cervical cytology and HPV testing for primary cervical cancer screening in Latin America: an analysis within the ESTAMPA study. *Lancet Reg. Health – Am.* **26**, (2023).
4. Definition of CIN 2 - NCI Dictionary of Cancer Terms - NCI. https://www.cancer.gov/publications/dictionaries/cancer-terms/def/cin-2 (2011).
5. Gao, G. *et al.* A novel RT-PCR method for quantification of human papillomavirus transcripts in archived tissues and its application in oropharyngeal cancer prognosis. *Int. J. Cancer J. Int. Cancer* **132**, 882–890 (2013).
6. Daskou, M. *et al.* WarmStart colorimetric LAMP for the specific and rapid detection of HPV16 and HPV18 DNA. *J. Virol. Methods* **270**, 87–94 (2019).
7. Barra, M. *et al.* Single-tube four-target lateral flow assay detects human papillomavirus types associated with majority of cervical cancers. *Anal. Biochem.* **688**, 115480 (2024).
8. Luo, L. *et al.* Visual detection of high-risk human papillomavirus genotypes 16, 18, 45, 52, and 58 by loop-mediated isothermal amplification with hydroxynaphthol blue dye. *J. Clin. Microbiol.* **49**, 3545–3550 (2011).
9. Wang, J. *et al.* Detection of 14 High-Risk Human Papillomaviruses Using Digital LAMP Assays on a Self-Digitization Chip. *Anal. Chem.* **93**, 3266–3272 (2021).

10. Zhang, Y. *et al.* Enhancing colorimetric loop-mediated isothermal amplification speed and sensitivity with guanidine chloride. *BioTechniques* **69**, 178–185 (2020).
