# Supplementary material for: One-hour extraction-free loop-mediated isothermal amplification HPV DNA assay for point-of-care testing in Maputo, Mozambique
Source: Nat Commun. 2025 Aug 7;16:7295. doi: 10.1038/s41467-025-62454-x (PMC12331945; doi:10.1038/s41467-025-62454-x)
Supplement: Supplementary file 2 — Reporting Summary [file 41467_2025_62454_MOESM2_ESM.pdf]

nature portfolio

Corresponding author(s): Rebecca Richards-Kortum
Last updated by author(s): Jul 21, 2025

Reporting Summary

Nature Portfolio wishes to improve the reproducibility of the work that we publish. This form provides structure for consistency and transparency in reporting. For further information on Nature Portfolio policies, see our Editorial Policies and the Editorial Policy Checklist.

Statistics

For all statistical analyses, confirm that the following items are present in the figure legend, table legend, main text, or Methods section.

n/a	Confirmed	
☐	☒	The exact sample size (*n*) for each experimental group/condition, given as a discrete number and unit of measurement
☐	☒	A statement on whether measurements were taken from distinct samples or whether the same sample was measured repeatedly
☐	☒	The statistical test(s) used AND whether they are one- or two-sided<br>*Only common tests should be described solely by name; describe more complex techniques in the Methods section.*
☒	☐	A description of all covariates tested
☒	☐	A description of any assumptions or corrections, such as tests of normality and adjustment for multiple comparisons
☐	☒	A full description of the statistical parameters including central tendency (e.g. means) or other basic estimates (e.g. regression coefficient) AND variation (e.g. standard deviation) or associated estimates of uncertainty (e.g. confidence intervals)
☒	☐	For null hypothesis testing, the test statistic (e.g. *F*, *t*, *r*) with confidence intervals, effect sizes, degrees of freedom and *P* value noted<br>*Give P values as exact values whenever suitable.*
☒	☐	For Bayesian analysis, information on the choice of priors and Markov chain Monte Carlo settings
☒	☐	For hierarchical and complex designs, identification of the appropriate level for tests and full reporting of outcomes
☒	☐	Estimates of effect sizes (e.g. Cohen's *d*, Pearson's *r*), indicating how they were calculated

Our web collection on statistics for biologists contains articles on many of the points above.

Software and code

Policy information about availability of computer code

Data collection	No software was used to collect data.
Data analysis	Graphpad Prism version 10.4.1 was used to analyze data and make figures. The Bio-Rad CFX Opus 96 Real-Time PCR System software (Bio-Rad CFX Maestro 2.3 version 5.3.022.1030) was used to analyze amplification curves. A custom MATLAB (version R2022b) code was used to analyze the amplification curves on the FAM (HPV16) channel for data collected on the Axxin T8-ISO and is available at 10.5281/zenodo.15930930.

For manuscripts utilizing custom algorithms or software that are central to the research but not yet described in published literature, software must be made available to editors and reviewers. We strongly encourage code deposition in a community repository (e.g. GitHub). See the Nature Portfolio guidelines for submitting code & software for further information.

Data

Policy information about availability of data

All manuscripts must include a data availability statement. This statement should provide the following information, where applicable:

- Accession codes, unique identifiers, or web links for publicly available datasets
- A description of any restrictions on data availability
- For clinical datasets or third party data, please ensure that the statement adheres to our policy

Source data are provided with this paper. All data generated in this study have been deposited in the Zenodo database under accession code 10.5281/zenodo.15865408

Research involving human participants, their data, or biological material

Policy information about studies with human participants or human data. See also policy information about sex, gender (identity/presentation), and sexual orientation and race, ethnicity and racism.

Reporting on sex and gender	Results from the testing of cervical samples are presented in this manuscript. The inclusion criteria for the protocols under which these samples were collected stated that the subject must have a cervix. Thus, anyone without a cervix was ineligible to be screened for cervical cancer, regardless of sex or gender. For the research presented in this manuscript, no attempt was made to generalize any information about the gender of the population providing the samples, thus gender was not tracked as part of this research.
Reporting on race, ethnicity, or other socially relevant groupings	Results from testing cervical samples are presented in this manuscript. For the research presented here, no attempt was made to generalize any information about the race or ethnicity of the populations providing the samples, thus race and ethnicity were not tracked.
Population characteristics	Results from the testing of cervical samples collected in Houston, Texas, USA are presented in this manuscript. The inclusion criteria for this protocol are included below:<br>1. People with a cervix 21 years of age or older.<br>2. Scheduled to undergo hrHPV testing at MD Anderson and The Harris Health System (LBJ Hospital) according to national and institutional guidelines at time of enrollment OR are anticipated to undergo a LEEP, ECC, or biopsy.<br>3. Willing and able to provide informed consent.<br>4. Able to perform protocol-required activities. Able to speak and read English or Spanish<br><br>Results from the testing of cervical samples collected in Mozambique and stored for future research are also presented in this manuscript. The inclusion criteria for the protocol under which the samples were collected and stored for future research included:<br>1. Subjects 30 – 49 years of age<br>2. Not currently pregnant<br>3. Subjects with a cervix (subjects who had undergone a total hysterectomy with removal of the cervix were not eligible)<br>4. Subjects presenting for care at clinics in Maputo City, Mozambique or Gaza Province, Mozambique<br>5. Willing and able to provide informed consent<br><br>The population characteristics relevant to the testing of the cervical samples are summarized in the manuscript.
Recruitment	For the cervical samples collected in Houston, TX, USA, eligible subjects scheduled to undergo high risk HPV testing at MD Anderson and The Harris Health System (LBJ Hospital) according to national and institutional guidelines at time of enrollment as well as eligible subjects who were anticipated to undergo a LEEP, ECC, or biopsy were invited to participate in this study.<br><br>For the samples collected in Mozambique and stored for future research, eligible subjects presenting to clinics for cervical cancer screening, voluntary family planning services, HIV care or other reasons in Maputo City or Gaza Province in Mozambique were invited to participate in a study which included the storage of their samples for future research. The research presented in this manuscript compares results from a new point of care HPV test (using the stored cervical samples) to the reference test that was used for patient treatment in Maputo City and Gaza Province when the samples were first collected and stored for future research.<br><br>For the research presented in this manuscript, no attempt was made to generalize any information about the population providing the samples.<br><br>For the Mozambique samples, samples with GeneXpert results were selected from the bank, and samples positive for the HPV types of interest (16, 18, 45) were selected as available.
Ethics oversight	The study protocols were reviewed and approved by IRBs at MDACC (2024-0020, 2020-0651), Harris Health System (24-05-3314), Comité Nacional de Bioética para a Saúde, Moçambique (IRB00002657), and Rice University (2024-388, 2021-48) prior to study initiation.

Note that full information on the approval of the study protocol must also be provided in the manuscript.

Field-specific reporting

Please select the one below that is the best fit for your research. If you are not sure, read the appropriate sections before making your selection.

- [x] Life sciences
- [ ] Behavioural & social sciences
- [ ] Ecological, evolutionary & environmental sciences

For a reference copy of the document with all sections, see nature.com/documents/nr-reporting-summary-flat.pdf

Life sciences study design

All studies must disclose on these points even when the disclosure is negative.

Sample size	Sample size calculation was not performed. Clinical samples tested were based on patient enrollment, banked sample availability and enrichment of HPV types relevant to our study.
Data exclusions	One sample evaluated in Mozambique produced an oscillatory fluorescence pattern that did not resemble amplification or a negative result and was omitted from analysis.
Replication	In all experiments except clinical sample testing, three, four, nine or ten replicates were performed. Bar graphs show mean results with standard deviation bars. After the assay was optimized, the performance of the assay was consistent across all experiments. We did not attempt to replicate clinical sample data, although results from both Houston and Mozambique were similar.
Randomization	Randomization was not relevant to this study, because it pertains to the development of a diagnostic test evaluated with de-identified samples and there is no intervention to randomize.
Blinding	For the optimization work, blinding was not relevant. Blinding was also not used with clinical samples in order to enrich the population with HPV-positive results, considering the low prevalence of HPV16/18/45, the types relevant to the LAMP test.

Reporting for specific materials, systems and methods

We require information from authors about some types of materials, experimental systems and methods used in many studies. Here, indicate whether each material, system or method listed is relevant to your study. If you are not sure if a list item applies to your research, read the appropriate section before selecting a response.

Materials & experimental systems

n/a	Involved in the study	
x		Antibodies
	x	Eukaryotic cell lines
x		Palaeontology and archaeology
x		Animals and other organisms
x		Clinical data
x		Dual use research of concern
x		Plants

Methods

n/a	Involved in the study	
x		ChIP-seq
x		Flow cytometry
x		MRI-based neuroimaging

Eukaryotic cell lines

Policy information about cell lines and Sex and Gender in Research

Cell line source(s)	American Type Culture Collection: SiHa (HTB-35), HeLa (CCL-2), MS751 (HTB-34), and C33A (HTB-31)
Authentication	Cell lines were authenticated with type-specific PCR as described in the methods section.
Mycoplasma contamination	Cell lines were not tested for mycoplasma contamination.
Commonly misidentified lines (See ICLAC register)	No commonly misidentified cell lines were used in this study.

Plants

Seed stocks	*Report on the source of all seed stocks or other plant material used. If applicable, state the seed stock centre and catalogue number. If plant specimens were collected from the field, describe the collection location, date and sampling procedures.*
Novel plant genotypes	*Describe the methods by which all novel plant genotypes were produced. This includes those generated by transgenic approaches, gene editing, chemical/radiation-based mutagenesis and hybridization. For transgenic lines, describe the transformation method, the number of independent lines analyzed and the generation upon which experiments were performed. For gene-edited lines, describe the editor used, the endogenous sequence targeted for editing, the targeting guide RNA sequence (if applicable) and how the editor was applied.*
Authentication	*Describe any authentication procedures for each seed stock used or novel genotype generated. Describe any experiments used to assess the effect of a mutation and, where applicable, how potential secondary effects (e.g. second site T-DNA insertions, mosiacism, off-target gene editing) were examined.*
